# Effects of simulated *in vitro* gastrointestinal digestion on antioxidant activities and potential bioaccessibility of phenolic compounds from *K. coccinea* fruits

**DOI:** 10.3389/fnut.2022.1024651

**Published:** 2022-12-15

**Authors:** Xiaochen Luo, Miaomiao Tian, Yingying Cheng, Caizhe Ji, Shende Hu, Hongnan Liu, Jun Lu, Jiali Ren

**Affiliations:** ^1^Hunan Key Laboratory of Forestry Edible Sources Safety and Processing, Central South University of Forestry and Technology, Changsha, China; ^2^College of Food Science and Engineering, Central South University of Forestry and Technology, Changsha, China; ^3^Institute of Subtropical Agriculture, Chinese Academy of Sciences, Changsha, China

**Keywords:** *Kadsura coccinea*, antioxidant capacity, simulate *in vitro* digestion, bioaccessibility, phenolic compounds

## Abstract

The changes of bioactive substances in peels and sarcocarps of three different varieties of *Kadsura coccinea* (Dahong, Fenhong, and Zihei) were evaluated by an *in vitro* simulation model. In this study, three varieties of *K. coccinea* fruits were digested *in vitro* to compare the changes and differences in antioxidant activity (DPPH, ABTS, FRAP) and bioaccessibility. The results showed that before digestion the highest content of total phenolics (TPC) (2.265 ± 0.127 mg GAE/g DW) and the highest content of flavonoids contents (TFC) (10.379 ± 0.093 mg GAE/g DW) were found in Fenhong fruit. The highest release of TPC and TFC after simulated gastric digestion was observed in the Dahong and Zihei sarcocarp, which increased by 98.7 and 20%, respectively. During oral and intestinal digestion, the content of bioactive compounds showed a decreasing trend. The radical scavenging ability of 1, 1-diphenyl-2-picrylhydrazyl (DPPH), [2,2-azino-bis(3-ethylbenzothiazoline) (ABTS)] sulfonic acid free radical scavenging ability, and ferric ion reducing antioxidant power (FRAP) results showed that the antioxidant capacity of *K. coccinea* fruits increased most significantly (26.9∼181%) in gastric digestion stage and then decreased after intestinal digestion stage. The recoveries of TPC and TFC were all increased after whole simulated digestion, as well as their bioaccessibility in sarcocarps was higher than those in peels, especially for the bioaccessibility of TPC and TFC in Dahong reached 50.53 and 48.19%, respectively. These results indicated that the peel and sarcocarp of *K. coccinea* had good antioxidant activity, with high bioaccessibility of phenolics could be a promising antioxidant source for the food industry.

## 1 Introduction

*Kadsura coccinea* (Lem.) A. C. Sm was a perennial evergreen vine, which is traditional Chinese medicine and belongs to the *Schisandra* family (1). Mainly distributed in Guizhou, Hunan, and other ethnic minority areas of China, it has proven to have antitumor (2), anti-HIV (3), lipid-lowering (4), antioxidation (5), NO inhibitor activity (6), antiviral, anti-inflammatory (7) and other pharmacological effects, which was considered as a treasured herbal medicine. The skin color of these varieties is varied with different varieties. The ripening of the sarcocarp is indicated by a unique scent. In addition, *K. coccinea* fruit is rich in nutrients and abundant polyphenols (8). Previous researchers measured the total polyphenols of *K. coccinea* and found that antioxidant activity in fruit was greater than that in other parts of the plant, and its nutritional components were analyzed by metabonomics proved that it contains organic acids, sugar alcohols, aliphatic acids, large number of amino acids and their derivatives, etc (9). Therefore, the nutritional value of *K. coccinea* has become a hot topic in recent years.

Plants contain a variety of phenolic compounds, which can be transformed and absorbed by the human body, but the absorption rate of polyphenols will change significantly after absorption in the digestive tract. During intestinal digestion, the complex mixture of polyphenols is degraded into single phenols, such as syringic acid, cinnamic acid, caffeic acid, and protocatechuic acid (10). Under the influence of intestinal temperature, reaction time, pH, human enzymes, and other factors, they were absorbed into the blood, liver, bile, and other parts of the final absorption and metabolism. *In vitro* simulated digestion experiments have been widely used in recent years to explore plant activity due to their easy control of experimental conditions and short and simple experimental cycles (11). Currently, the effect of the human gut environment on the biological and chemical properties of the antioxidant capacity of *K. coccinea* is unknown. In this study, antioxidant activity and phenolic compounds *in vitro* simulate the digestion of three varieties of *K. coccinea* fruits obtained from Tongdao county of Hunan Province in China were measured for the first time. By simulated *in vitro* digestion method, the contents of total polyphenols and flavonoids in different parts and antioxidant activities were also evaluated, as well as their recovery and bioaccessibility. The results of this study provide theoretical support for its future application in functional food and health products. The experimental ideas and structure of present paper are showed in Supplementary Figure 1.

## 2 Materials and methods

### 2.1 Materials and reagents

Three varieties of *K. coccinea* fruit were collected from Tongdao County, Hunan Province, China (26°54′ N, 109°42′ E), including Zihei (ZH), Fenghong (FH), and Dahong (DH). Folin-Ciocalteau and Gallic acid were purchased from Shanghai Yuanye Biotechnology Co., Ltd. Human saliva α-Amylase (300–1,500 U/mg protein, CAS 9000-90-2), pepsin from porcine gastric mucosa (0.7 FIP-U/mg for biochemistry EC 3.4.23.1), pancreatin from porcine pancreas (4X UPS, CAS 8049-47-6), and bile salt (CAS 8008-63-7) were purchased from Sigma Company of the United States. Water Soluble Vitamin E (Trolox), 1, 1-Diphenyl-2-Picrylhydrazyl,(DPPH) were purchased from TIC Co., LTD. 2,2′-Azino-BIS(3-ethylbenzothiazoline-6-sulfonic acid), (ABTS) was purchased from Hefei Bomei Biological Co., Ltd.

### 2.2 Experimental instruments

The instruments used in this study were as follows: UV1800 UV-Vis Spectrophotometer Shanghai Precision Scientific Instrument Co., Ltd., AuY220 Electronic Analytical Balance Japan Shimadzu Corporation; ZHWY-2102C Constant Temperature Cultivation Shaker Shanghai Zhi Cheng Analytical Scientific Instrument Co., Ltd., Sorvall LYNX6000 refrigerated centrifuge, American Thermo fisher company.

### 2.3 Methods

#### 2.3.1 Determination of physical indicators

Physical indicators of different varieties of *K. coccinea* were determined, and five fruits of each variety were randomly selected. A vernier caliper is used to measure the horizontal and vertical diameters, and the ratio of the vertical diameter to the horizontal diameter is the fruit shape index. Peeling the fruits into single petals, calculating the number and weight of single petals, and weighing 10 single petals of known mass were done manually. Then, the edible rate is calculated according to the following formula: edible rate = (total weight–seed weight–tare weight)/ total weight.

#### 2.3.2 Extraction of TPC and TFC by *in vitro* chemical method

The extracting of phenolic compounds by the chemical method was slightly modified according to the Lu et al. (12) method. The peel and sarcocarp were separated manually and dried in a drying oven at 45°C for 30 h. After that, the dried materials were pulverized and passed through a 60-mesh sieve to obtain experimental samples, then 1 g of the prepared sample was mixed well with 20 ml of 60% ethanol (containing 0.1% hydrochloric acid), and extracted for 1 h under ultrasonic extraction at 40°C and 500 W power. Then, the supernatant was collected after the sample was centrifuged at 4,000 r/min for 8 min, at room temperature. The precipitate was added with 10 ml of 60% ethanol to repeat the above procedure, and the extracts of two times were combined for further analysis.

#### 2.3.3 *In vitro* simulated digestion

*In vitro* digestion was carried out as the previous description of the INFOGEST protocol given by Minekus et al. (13) and Gullon et al. (14) with slight modification. Three parts of the phase including oral, gastric, and intestinal were used for simulated digestion *in vitro*, and digestive juice of three stages is collected separately. All enzyme solutions in the experiment need to be prepared immediately.

To simulate oral digestion, 1 g of the sample was mixed with 20 ml of distilled water and 1 ml of 100 U/ml α-amylase solution (the amylase solution was prepared with 1 mM CaCl_2_ and adjusted to pH 6.9 with 1 M NaHCO_3_). The contents of active substances and their antioxidant activity were determined after the mixture was reacted at 37°C for 5 min.

After oral digestion was completed, gastric digestion was simulated by adjusting the pH of the solution to 2 with hydrochloric acid (6 M) and then adding 1 ml of pepsin solution (0.108 g enzyme dissolved in 10 ml 0.1 M hydrochloric acid). The reaction was performed in a shaking water bath at 37°C, 50 rpm for 2 h, then the samples were refrigerated and centrifuged, and the TPC, TFC, and antioxidant activity were determined.

After the oral and gastric digestion stages, the pH was raised to 7 for partial inactivation of pepsin or pH of 8 for complete inactivation. The post-gastric digestion solution was adjusted to pH 7 with NaOH (6 M), then 2.5 ml of pancreatin (80 mg of enzyme dissolved in 10 ml of 0.5 M NaHCO_3_), and 2.5 ml of bile salt mixture (500 mg bile salts were dissolved in 10 ml 0.5 M NaHCO_3_) were added, and the reaction was continued for 2 h in a shaking water bath at 37°C, 50 rpm. After that, the digested sample solution was transferred to a dialysis bag to dialyze to sodium chloride (10 mM) overnight at 37°C. All solutions were maintained in an ice bath during the entire period of gastrointestinal digestion processes prior to gradual addition.

When the above process was completed, the solution remaining in the dialysis tubing was taken as the IN sample, representing material retained in the gastrointestinal tract (colon available), and the dialysate as the OUT sample (serum available). The IN sample was centrifuged at 8,000 *g* for 12 min at 4°C to obtain the soluble fraction (CSF) and the particulate fraction (PF). After extraction with acidified ethanol, both fractions were stored at 4°C for the active substance content and antioxidant activity of the PF were determined.

#### 2.3.4 Determination of total phenolics (TPC) and flavonoids contents (TFC)

The total phenolic content was determined by the Folin-phenol reagent colorimetric method (15). Briefly, 1 ml of sample solution was added with distilled water to 23 ml, then 500 μl of Folin reagent and 300 μl of 10% sodium carbonate solution were added. The absorbance was measured at a wavelength of 760 nm after the mixed solutions were reacted for 30 min. The total phenolics content in the sample was calculated according to the standard curve of gallic acid and the results were expressed as milligram gallic acid equivalents per gram dry weight sample (mg GAE /DW).

The total flavonoid content was measured according to the reference reported by Zielinski et al. (16). Catechin was used as the standard, and a standard curve was drawn with the catechin concentration as the abscissa and the absorbance at 510 nm as the ordinate. Briefly, 250 μl of sample solution was mixed with 2,720 μl of 30% ethanol solution and 120 μl of 0.5 mol/L sodium nitrite solution to react for 5 min. Then, 120 μl of 10% aluminum chloride solution was added for another 5 min of reaction. Finally, 800 μl of 1 mol /L sodium hydroxide was added and the absorbance was measured at 510 nm with 30% ethanol as blank. The content of flavonoids in the sample was calculated according to the standard curve of catechin and expressed as milligram catechin equivalent per gram dry extract sample (mg CE/DW).

#### 2.3.5 Determination of antioxidant activities

Three assays of antioxidant activities were used to evaluate the comprehensive antioxidant activity of *K. coccinea*. The 1,1-diphenyl-2-picrylhydrazyl (DPPH) radical scavenging activity of samples was determined according to the procedure of Gorjanović et al. (17) with slight modifications. Briefly, 300 μl of sample solution and 1.9 ml of 0.094 mM DPPH solution were mixed and kept in the dark place at room temperature for 30 min reaction before the absorbance was read at 517 nm. The results were reported as μmol Trolox equivalents per gram dry weight of the sample (μmol TE/g DW), using 80% methanol as the blank sample.

The 2, 2′-azino-bis-3-ethylbenzothiazoline-6-sulfonic acid (ABTS) radical scavenging activity of samples was performed according to the method of Oh et al. (18) with slight modifications. Briefly, 7.4 mM ABTS stock solution was mixed with equal quantities of 2.4 mM potassium persulfate to make the ABTS solution, and the mixture was incubated at room temperature for 12–16 h in the dark. Then, the solution was diluted with ethanol to obtain an absorbance of 0.70 ± 0.02 at 734 nm. A total of 1 ml of sample solution was mixed with 4.0 ml of ABTS solution. The mixture was placed in the dark at room temperature for 6 min before the absorbance was read at 734 nm. The blank sample was replaced by 80% methanol, and the results were reported as μmol Trolox equivalents per gram dry weight of the sample (μmol TE/g DW).

The ferric ion-reducing antioxidant power (FRAP) of samples was carried out following the procedure of Lu et al. (19) with slight modifications. Briefly, 2.5 ml of TPTZ (10 mM in 40 mM HCl) was mixed with equal quantities of 20 mM FeCl_3_ and 25 ml of acetate buffer (0.3 M, pH 3.6). Then, 900 μl of sample solution was mixed with 2.7 ml of TPTZ solution and 270 μl of deionized water. The mixture was reacted at room temperature for 30 min before the absorbance was read at 595 nm using Trolox as a positive control, taking 70% methanol as the blank sample. The antioxidant capacity was expressed as μmol Trolox equivalents per gram dry weight of the sample (μmol TE/g DW).

#### 2.3.6 Determination of recovery rate and bioaccessibility

The recovery rate can be obtained by comparing the TPC and TFC before and after *in vitro* simulated digestion, and it was calculated by Formula (1). Bioaccessibility was defined as the percentage of active compounds dissolved in CSF after intestinal dialysis steps. Therefore, this indicator represents the proportion of active compounds that can be absorbed into the circulation of the human system (serum). It can be obtained by comparing the total amount of bioactive compounds in the post-dialysis OUT portion (serum available) and pre-dialysis sample (IN + OUT) during intestinal digestion, following Formula (2).


(1) $$R\%=\frac{PC_{DF}}{PC_{TM}}\times 100$$

(2) $$BI\%=\frac{PC_{S}}{PC_{DF}}\times 100$$

R is the recovery of TPC or TFC after each digestion stage. BI is the bioaccessibility of TPC or TFC that is related to the percentage of bioactive compounds. PC_*DF*_ is the TPC or TFC in the digested fraction (soluble fraction CSF + precipitated PF) after each digestion stage (oral, stomach, and intestine). PC_*TM*_ is the TPC or TFC in the experimental matrix before digestion. PC_*S*_ is the TPC or TFC in the OUT sample after the dialysis at intestinal digestion. PC_*DF*_ is the TPC or TFC in the total digest sample (IN+OUT) after the dialysis phase.

#### 2.3.7 Data analysis

The experiments were performed in triplicate and the data were expressed as mean ± standard deviation. One-way analysis of variance (ANOVA) and (two-way ANOVA) were performed and the significant differences in the results were determined by Tukey’s test at a 5% significance level. Duncan’s method was used to make multiple comparisons between data differences. SPSS 24.0 was used for statistical analysis and Origin software was used for drawing.

## 3 Results and discussion

### 3.1 Physical quality of fruits of different varieties of *K. coccinea*

Table 1 shows the results of the physical indicators of the three varieties of *K. coccinea* fruits. Among the three varieties, ZH had the highest single fruit weight and shape index with the number of single petals from 38 to 54. The single fruit weight of the DH is the lowest, but its edible rate was the highest with a value of 47.3%, which was significantly higher than that of the ZH variety (40.4%). This was probably due to the ZH having a thicker peel and relatively less sarcocarp. The fruit shape of each variety was spherical, and there was no significant difference in the fruit shape index.

**TABLE 1:** Physical quality of *Kadsura coccinea.*

Variety	Shape index	Single fruit weight	Single petals	Edible rate	Pericarp thickness
ZH	1.03 ± 0.09^a^	416.1 ± 21.85^a^	38–54	40.4%^b^	thick
DH	0.93 ± 0.02^a^	376.0 ± 50.22^b^	38–43	47.3%^a^	moderate
FH	1.01 ± 0.16^a^	401.9 ± 42.50^a^	38–57	46.7%^a^	thin

ZH, Zihei; DH, Dahong; FH, Fenhong. Results are expressed as the mean ± SD (*n* ≥ 5), different letter means with different letters within the same column indicate statistical differences (*p* < 0.05). Data with different lowercase letters in same column indicate significant differences (*P* < 0.05).

### 3.2 The TPC, TFC, and antioxidant activities before simulated *in vitro* digestion

The determination results of TPC, TFC, and antioxidant activities in the peel and sarcocarp of the three varieties of *K. coccinea* extracts are shown in Table 2. The content of TPC, TFC, and antioxidant capacity of different species and parts of *K. coccinea* were analyzed by the two-way variance method.

**TABLE 2:** Content of TPC and TFC and antioxidant activity of *Kadsura coccinea* before digestion.

	Variety	TPC (mg GAE/g DW)	TFC (mg CE/g DW)	DPPH (μ mol TE/g DW)	ABTS (μ mol TE/g DW)	FRAP (μ mol TE/g DW)
Peel	ZH	1.957 ± 0.093^b^	5.670 ± 0.219^c,***^	5.535 ± 0.074^a,***^	9.100 ± 0.075^b,*^	85.607 ± 2.960^b,***^
	DH	1.831 ± 0.043^c,***^	4.196 ± 0.110^d,***^	5.332 ± 0.037^a,***^	8.951 ± 0.136^b^	72.600 ± 5.709^c^
	FH	2.265 ± 0.127^a,*^	5.321 ± 0.165^c,**^	5.566 ± 0.015^a,***^	11.606 ± 0.090^a,***^	95.923 ± 1.480^a,***^
Sarcocarp	ZH	1.878 ± 0.037^c^	10.379 ± 0.093^a,***^	4.434 ± 0.013^b,***^	9.024 ± 0.064^b,*^	69.880 ± 1.441^d,***^
	DH	1.826 ± 0.049^c,***^	8.463 ± 0.187^b,***^	4.252 ± 0.031^b,***^	8.860 ± 0.090^b^	68.861 ± 1.081^d^
	FH	1.844 ± 0.073^c,*^	5.920 ± 0.140^c,**^	4.394 ± 0.019^b,***^	8.197 ± 0.231^c,***^	68.352 ± 1.360^d,***^

ZH, Zihei; DH, Dahong; FH, Fenhong. Results are expressed as the mean ± SD (*n* ≥ 5), different letter means with different letters within the same column indicate statistical differences (*p* < 0.05). *Indicates statistical significance in different parts of the same variety (*show *p* < 0.05, **show *p* < 0.01,***show *p* < 0.001). Data with different lowercase letters in same column indicate significant differences (*P* < 0.05).

The TPC ranged from 1.826 to 2.265 mg GAE/g DW. Among them, the FH peel showed the highest level of TPC with a value of 2.265 ± 0.127 mg GAE/g DW, which was 1.24 times that of the DH peel with the lowest content. However, there was no significant difference in TPC in the sarcocarp of the three varieties. In addition, the TPC in the peel was higher than that of their corresponding sarcocarp except for Dahong. Especially for FH, which had the greatest difference as the TPC in the peel was 1.23 times that in the sarcocarp.

A similar trend was found in the TFC. ZH peel had the highest value (5.670 ± 0.219 mg GAE/g DW), which was 1.35 times the Dahong variety with the lowest content. The difference in sarcocarp among the three varieties also showed significance at level (*p* < 0.001). The TFC in ZH sarcocarp was 10.379 ± 0.093 mg GAE/g DW, which was 1.75 times that of the FH sarcocarp with the lowest content. In addition, compared with TPC, the TFC in the sarcocarp is much higher than that in the peel with probably two-folds. This was due to the dry sarcocarp being more concentrated after drying than the dry peel did, resulting in a corresponding increase in the flavonoid content of the sarcocarp. Sun et al. (5) extracted total phenols from the peel and sarcocarp of *K. coccinea* and found that the TPC of the peel extract was significantly higher than that of the sarcocarp and the TPC of extracts (by methanol, acetone, water, and formic acid) from *K. coccinea* peel tissues (179 1.95 μg/ml GAE).

For antioxidant activities, there was no significant difference among the DPPH radical ability of the three varieties of peel and sarcocarp, and the DPPH scavenging abilities of peel extracts were all higher than their corresponding sarcocarp extract. A similar tendency was found in FRAP and ABTS radical scavenging ability as the peel extract possessed higher FRAP and ABTS values than that of their corresponding sarcocarp. All fruit parts of ZH varieties showed high values of DPPH. It was noted that the strongest antioxidant activity among these three cultivars was found in their peel. This is most likely because the polyphenols in the peel are much higher than that in the sarcocarp, and the biological antioxidant activity is closely related to these bioactive components.

### 3.3 The effect of simulated *in vitro* digestion on the active substance content and antioxidant activity

#### 3.3.1 Changes in TPC during simulated *in vitro* digestion

**FIGURE 1:**
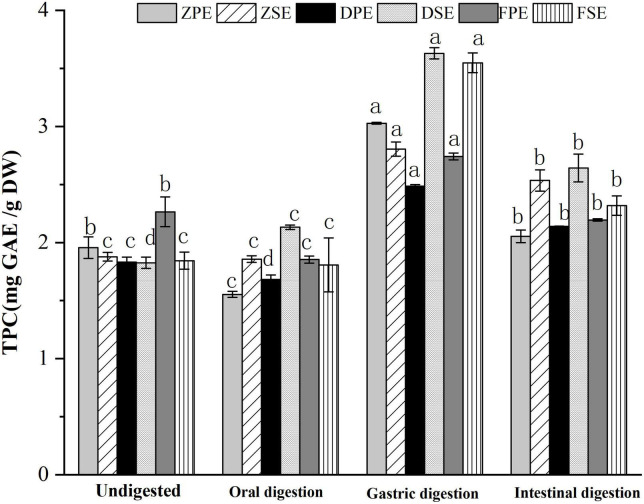
The changes of TPC in the peel and sarcocarp of *Kadsura coccinea* fruits during simulated *in vitro* digestion. Values are presented as means ± SD (*n* = 3), (*p* < 0.05). ZPE, Zihei peel extract; DPE, Dahong peel extract; FPE, Fenhong peel extract; ZSE, Zihei sarcocarp extract, DSE, Dahong sarcocarp extract; FSE, Fenhong sarcocarp extract.

The changes of TPC in the peel and sarcocarp of *K. coccinea* during simulated *in vitro* digestion are shown in Figure 1. After oral digestion, the TPC in the peel and sarcocarp of *K. coccinea* decreased to different degrees. Among them, ZPE showed the greatest decrease from 1.957 to 1.552 mg GAE/g DW, which means 20.7% of TPC was decreased. Followed by FSE, the TFC was decreased by 12% from 2.265 to 1.854 mg GAE/g DW after digestion. Compared with the peel, the decreased degree of polyphenols in the sarcocarp was smaller and the change was not obvious. After simulated gastric digestion, the TPC of the three varieties increased significantly. The largest change was found in ZPE, which increased by 55% from 1.957 to 3.027 mg GAE/g DW. The amount of TPC released from sarcocarp increased evidently, DSE showed the greatest increase from 1.826 to 3.629 mg GAE/g DW, for a 98.7% of improvement. After intestinal digestion, the TPC of peel and sarcocarp showed a significant (*p* < 0.05) downward trend (9.6–34%) compared with the TPC after the oral digestion stage. Similar results were also found in grape (20), apple (21), and blueberry (22), with the largest release of polyphenols during gastric digestion, while most of the polyphenols will be degraded during intestinal digestion. Previous studies have reported 14 substances with antioxidant activity from *K. coccinea* peel, such as salidroside, syringic acid (23), and protocatechuic acid (10). The valence bond was connected to the polysaccharide in the cell wall. In oral and gastric digestion, due to the acidic environment of the solution, the pH decreases resulting in the polysaccharide and lignin ester bonds, so the release amount will increase. Besides, the procyanidin would be hydrolyzed, increasing the water solubility of phenolic substances. However, during the simulated intestinal digestion, the intestinal juice was in a medium or weak alkaline environment, so the polyphenols were unstable and then degraded.

#### 3.3.2 Changes in TFC during simulated *in vitro* digestion

**FIGURE 2:**
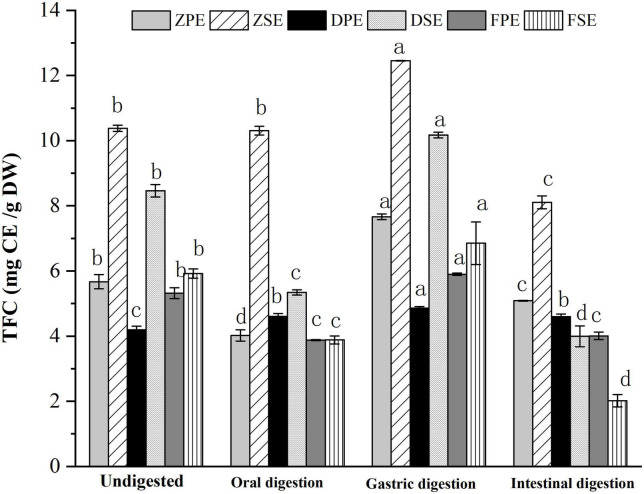
The changes of TFC in the peel and sarcocarp of *Kadsura coccinea* fruits during simulated *in vitro* digestion. Values are presented as means ± SD (*n* = 3), (*p* < 0.05). ZPE, Zihei peel extract; DPE, Dahong peel extract; FPE, Fenhong peel extract; ZSE, Zihei sarcocarp extract, DSE, Dahong sarcocarp extract; FSE, Fenhong sarcocarp extract.

The changes of TFC in the peel and sarcocarp of *K. coccinea* in the simulated digestion process are shown in Figure 2. After oral digestion, the content of flavonoids of the three varieties was decreased compared to their corresponding flavonoid before digestion except for ZSE. Among the three peels, the ZPE showed the largest decrease of 29.14% from 5.670 to 4.018 CE/g DW. The DSE and FSE decreased significantly by 37 and 34%, respectively. After gastric digestion, the TFC of all varieties of *K. coccinea* increased significantly more than the TFC after the oral digestion stage, among which, the increase of ZPE and ZSE was the most prominent, for 35.2 and 20%, respectively. The flavonoid content in DPE and DSE increased by 15.8 and 20.2%, respectively, while the flavonoid content in PPE and PSE increased by 10.9 and 15.8%, respectively. During the intestinal stage, the FSE decreased most obviously by 70.6% from 6.854 CE/g DW at the initial stage of intestinal digestion to 2.017 CE/g DW. The TFC changes were similar to the changes of TPC in that most of them were increased in the gastric stage and decreased in the intestinal stage. The present result consisted of the report on citrus (24) and hawthorn (25). This may be related to the flavonoid glycosides released from the complex system of food as the hydrolysis by the hydrochloric acid in gastric juice, or the bound phenolics were converted into free phenol. Consequently, the TPC and TFC showed higher levels than the oral stage. Whereas under the effect of intestinal alkaline environment in intestinal juice, the flavonols were unstable and prone to degradation, as rich anthocyanins in *K. coccinea* (26), most of them exist in the form of glycosides (cyaninin-3-xylo-rutin, delphinin-3-xylo-rutin, etc.) with ester bonds connected between the glycosides are easily hydrolyzed by trypsin in an alkaline environment, therefore, the relative lower level of TPC and TFC were observed at the intestinal stage.

#### 3.3.3 Changes in antioxidant activity during simulated *in vitro* digestion

**FIGURE 3:**
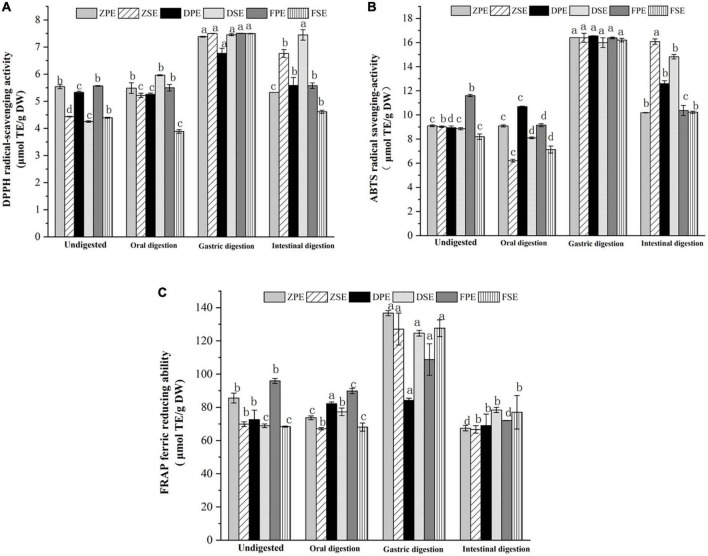
Effects of antioxidant activity DPPH **(A)**; ABTS **(B)**; and FRAP **(C)** in simulated digestion of *Kadsura coccinea*. fruits. Values are presented as means ± SD (*n* = 3), (*p* < 0.05). ZPE, Zihei peel extract; DPE, Dahong peel extract; FPE, Fenhong peel extract; ZSE, Zihei sarcocarp extract, DSE, Dahong sarcocarp extract; FSE, Fenhong sarcocarp extract.

Three antioxidant activity methods were employed to evaluate the changes in antioxidant activity during simulated *in vitro* digestion. The changes in the DPPH scavenging ability of *K. coccinea* peel and sarcocarp are shown in Figure 3A. It was found that oral digestion had no obvious effect on the DPPH scavenging ability of the three varieties of *K. coccinea*. However, after gastric digestion, the DPPH of ZSE, DSE, and FSE increased by 69.1, 75.3, and 70.6%, respectively, compared with the indigestion sarcocarp. After intestinal digestion, the DPPH of all samples decreased compared with the gastric digestion, especially for the FSE had most reduced by 41.4% from 7.495 to 4.394 μmol TE/g DW. The ZSE and DSE did not show a significant decrease which may be related to the changes in TPC and TFC in the different digestion processes. A study by Timur et al. (27) indicated that the DPPH antioxidant activity of European cranberry decreased significantly during simulated digestion, which was due to the decomposition of fruit active metabolites by intestinal microbes during digestion.

The changes in ABTS scavenging ability exhibited a similar tendency with the above antioxidant activity assay and the results are shown in Figure 3B. In oral digestion, the ABTS scavenging ability of the peel and sarcocarp of the three varieties of *K. coccinea* also decreased slightly, and the antioxidant activity increased significantly after gastric digestion. The ABTS scavenging ability of the peels of ZH, DH, and FH displayed 80.3, 84.8, and 41.2%, respectively, increases in the peel sample before the digestion. Meanwhile, 81.7, 80.4, and 97.7% of increases were also found in their sarcocarp gastric digestion sample. However, all of them showed a descending trend in the intestinal digestion stage, with a decreasing rate from 5.3% (ZSE) to 36.9% (FSE).

Figure 3C showed the changes in FRAP antioxidant activity during the simulated digestion *in vitro* process. After oral digestion, the FRAP antioxidant activity of ZH and FH varieties decreased slightly, but Dahong showed a slight increase. After gastric digestion, the FRAP values of *K. coccinea* sarcocarp were significantly increased, and ZPE showed the greatest change of 59.7% from 85.607 μmol TE/g DW in indigestion to 136.714 μmol TE/g DW. The FRAP value of the sarcocarp in ZH, DH, and FH also increased by 95.8, 118.6, and 181%, respectively, to their corresponding undigested sarcocarp. However, after intestinal digestion, the FRAP values of the peel and sarcocarp all decreased significantly, with the decline rates from 18.1 to 50.6%. Especially, the ZPE value showed the most significant change from 136.714 μmol TE/g DW in gastric digestion to 67.448 μmol TE/g DW in intestinal digestion.

From the above results, we can conclude that the antioxidant capacity of *K. coccinea* peel and sarcocarp measured by the three methods showed a similar pattern during the simulated digestion process *in vitro*, as there was no significant change in oral digestion, but it increased significantly after gastric digestion and then decreased significantly during intestinal digestion. These results were consistent with that observed in some fruits like European cranberry (28) and raspberry (29). Their antioxidant activities showed no obvious change after oral digestion and reached the maximum value after gastric digestion, then the antioxidant activity showed a downward trend during intestinal digestion. These results could be explained by their changes in TPC and TFC contents during the simulated digestion, as the antioxidant capacity was highly related to active substances like phenolics and flavonoids. In different stages of digestion, the number of polyphenol antioxidants released from complicated food systems has differences. Such as in the oral stage, due to the effect of the degradation reaction of the microbes and enzymes, catechin and epicatechin conversion (30), but due to the short contact time with enzyme, the polyphenols release a quantity that is significantly lower than the subsequent stage of stomach and intestine, and is under the stimulus of acidic gastric juice (31). The unstable decomposition of procyanidins in plant cell walls increases the content of anthocyanins I, thus enhancing their antioxidant activity. For example, apple (32) showed an antioxidant capacity of free soluble polyphenols after gastrointestinal digestion and dialysis, and the release of polyphenols mainly occurred in gastric digestion. Large amounts of polyphenols (about 65% of phenols and flavonoids) are released at this stage. During intestinal digestion at neutral or slightly alkaline pH, most polyphenols are lost during incubation with pancreatic bile salts, or most phenolic compounds have been degraded or converted into new compounds. Previous studies indicated that the acid-base change of the medium can cause the release of phenolic substances, which was due to the deprotonation of the hydroxyl group in the aromatic ring (21), which helps to improve the antioxidant activity of phenolic compounds. However, the ABTS assay (conducted at a buffered pH = 7) could be more appropriate to evaluate the intestinal digesta with pH of 7–7.5. Xochitl et al. (28) explored the antioxidant activity of 12 Plum (*Spondias purpurea* L.). Ecotypes through simulated *in vitro* digestion and found that in the stage of intestinal digestion, the content of polyphenols, flavonoids, and antioxidant activity showed a high correlation.

### 3.4 Recovery rate of TPC and TFC during simulated digestion *in vitro*

**FIGURE 4:**
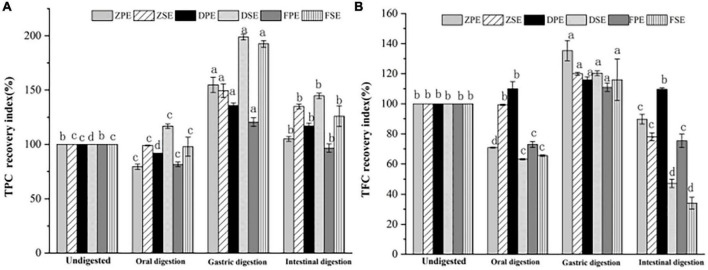
The recovery rate of TPC **(A)** and TFC **(B)** after simulated *in vitro* digestion of different stages. Values are presented as means ± SD (*n* = 3), (*p* < 0.05). ZPE, Zihei peel extract; DPE, Dahong peel extract; FPE, Fenhong peel extract; ZSE, Zihei sarcocarp extract, DSE, Dahong sarcocarp extract; FSE, Fenhong sarcocarp extract.

The recovery rates of TPC and TFC after simulated oral, gastric, and intestinal digestion *in vitro* are shown in Figure 4A, after oral digestion, the recovery rate of polyphenols in *K. coccinea* peel was relatively low, among them, the ZPE was the lowest, for only 79.33%. Meanwhile, the polyphenol content of sarcocarp was not obviously different from that in the undigested sample. After gastric digestion, the recovery rates of polyphenols showed a significant upward trend. For peel, the recovery index ranged from 120.53% for PPE to 154.81% for ZPE. The recovery rate of sarcocarp was higher than that of peel. Among them, the recovery rate of DSE was the highest with a value of 198.71%. After intestinal digestion, the recovery index of polyphenols in *K. coccinea* peel and sarcocarp all decreased sharply with the value ranging from 125.92 to 144.64%.

Figure 4B shows the recovery rate of TFC after simulated digestion of different stages *in vitro*. A similar tendency with the TPC recovery index was found in three simulated digestion phases. After oral digestion, the recovery rates of flavonoids from *K. coccinea* peel and sarcocarp decreased to varying degrees and then increased significantly after gastric digestion from 110.96 to 135.30%. After intestinal digestion, the recovery rate of peel and sarcocarp decreased significantly from (29.4–94.2%). Moreover, all the peels showed a greater recovery rate than that of sarcocarp.

The present results indicated that the oral digestion stage affects the content of biologically active substances in the peel and sarcocarp of *K. coccinea* to varying degrees. After gastric digestion, the recovery rate of TPC and TFC increased significantly. The effects of GI digestion of *U. dioica* leaves on the TPC, TFC, and antioxidant activity demonstrated that digested leaves retained greater TPC and TFC, and antioxidant activity (33). Under the action of gastric simulated peristalsis, pepsin, and pH, the particle size of chyme of phenolic polymers decreased, which was beneficial to the release of phenolic acids (34). Besides, under the action of proteases, polysaccharides, peptides, and other nutrients, some phenolic substances that bound covalently or non-covalently to proteins are released. Therefore, the recovery of TPC after gastric digestion increased significantly. Due to the weak alkaline or neutral environment during intestinal digestion, bile, pancreatic enzymes and the interaction of food in the intestine reduce the content of active substances (35). After being uptake by small intestinal epithelial cells, the active substances are metabolized and secreted in the cells and eventually enter the blood. Phenolics that are undigested in the small intestine are transferred to the colon, where they are reabsorbed by the microbial community through fermentation. The same plasma concentration also affects the activation and release of phenolic antioxidants. For example, the conversion and absorption of procyanidins are mainly through depolymerization into epicatechin units, which are absorbed in the small intestine and directly fermented by intestinal microbiota to form hydroxycinnamic acid series (36). The change law of TPC and TFC in pears during simulated digestion *in vitro* was studied (37), and it was found that the release of polyphenols from three different varieties of pears during simulated intestinal digestion *in vitro* increased compared with the initial stage of gastric digestion. The maximum amount of polyphenols released by Yali, Mili, and Fragrant pears were 1.83, 1.99, and 2.58 times, respectively, greater than the amount at 0 h of gastric digestion. However, the weak alkaline or neutral environment during intestinal digestion, bile, pancreatic enzymes and the interaction of food in the intestine reduce the content of active substances (35).

### 3.5 Bioaccessibility of *K. coccinea* polyphenols and flavonoids

**FIGURE 5:**
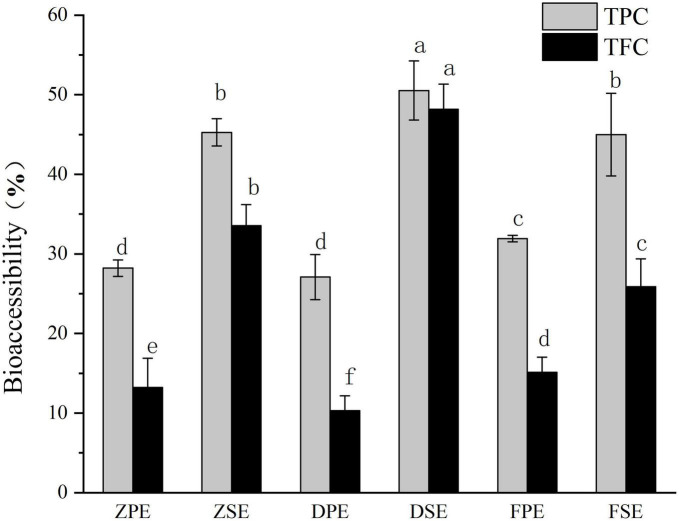
The bioaccessibility of polyphenols and flavonoids in *Kadsura coccineas* fruits after simulated *in vitro* digestion. Values are presented as means ± SD (*n* = 3), (*p* < 0.05). ZPE, Zihei peel extract; DPE, Dahong peel extract; FPE, Fenhong peel extract; ZSE, Zihei sarcocarp extract, DSE, Dahong sarcocarp extract; FSE, Fenhong sarcocarp extract.

Bioaccessibility refers to the extent to which certain active substances are absorbed by the circulatory system after consumption. The bioaccessibility of TPC and TFC in *K. coccinea* was between 10.32 and 50.53% (Figure 5). For the peel, the highest TPC bioaccessibility was 31.93% for the Fenhong variety, followed by 28.22% for the Zihei variety, and 27.10% for the Dahong variety, and the total flavonoids in the peel showed the same trend. The bioaccessibility of polyphenols and flavonoids in *K. coccinea* sarcocarp was relatively higher, among which the DSE had the highest bioaccessibility, respectively, reaching 50.53 and 48.19%, and the lowest was FSE, for 44.98 and 25.88%, respectively. The present results were lower than the index value (64.02%) of *Punica granatum L*. (12) and higher than that of *Persimmon* with the index value (5.31% for free phenolics and 21.54% for bound phenolics). This difference is probably due to the polymerization degrees, spatial configuration, and specific group, as in the process of simulated digestion *in vitro*. In organisms, polyphenols and dietary fiber are connected by covalent bonds and non-covalent bonds (hydrogen bonds, hydrophobic interactions, electrostatic interactions, etc.). Ionic strength, temperature, polyphenols, and dietary fiber structure all affect their release. The low pH and pepsin action during gastric digestion release phenolic compounds that bind to carbohydrates, thus making these bioactive compounds exhibit high bioavailability (36).

Moreover, the absorption and utilization rate of plant active substances is also related to the molecular weight of the substance. The smaller the molecular weight of a substance, the higher its absorption in the intestinal epidermis, blood, liver, and gallbladder. Some phenolics become unstable small molecules after simulated digestion, such as gallic acid (38). The most important place for flavonoids, such as proanthocyanidins, which can only be released when depolymerized into anthocyanins to be absorbed in the human body is not the intestinal tract. Due to the large molecular weight of theaflavins in tea leaves, the recovery rate is low which can be observed in urine according to Scalbert et al. (38). Active substances in food can only be absorbed by the gastrointestinal mucosa if they are released from the food matrix to which they are bound. The covalent bonding of cell wall polysaccharides and phenolic acids prevents phenolic acids from being cleaved by biological enzymes in the human body (39). The nature of the food itself, such as the degree of binding with the matrix, and the contained nutrients will affect the utilization of phenolic substances. Dietary cellulose and pectin can well promote the release of anthocyanins and phenolic acids in the intestine. Pectin can prevent the solubilization of food in gastric juice (40), allowing it to be released in large quantities in the intestinal phase. *K. coccinea* contains a large amount of flavonoids and phenolic acids, such as anthocyanins and PHBA (p-hydroxybenzoic acid), which can achieve high bioaccessibility, and these characteristics demonstrate that *K. coccinea* is a potential raw material for processing into functional food.

## 4 Conclusion

The present study reported the effects of simulated *in vitro* digestion on bioactive compounds (phenolics and flavonoids) and antioxidant capacity (DPPH, ABTS, FRAP) of peel and sarcocarp from three typical *K. coccinea in vitro* for the first time. The results proved that the TPC, TFC, and antioxidant activity in all the peel and sarcocarp of the *K. coccinea* fruits almost showed no significant change under the oral stage, whereas the TPC and TFC exhibited a greater release in the simulated gastric digestion phase (10.9∼98.7%), and their antioxidant activities were increased correspondingly (26.9∼181%) as well, but they were significantly decreased in the following intestinal digestion stage. However, after these three processes were finished, there still showed higher levels of TPC, DPPH, and ABTS radical scavenging activity than that of indigestion *K. coccinea* fruits. These results are probably in line with the release content of total phenolics and total flavonoids. Additionally, under the simulated digestion, these three varieties of *K. coccinea* also showed a higher recovery rate and moderate bioaccessibility for total phenolics and total flavonoids. The results of this study are helpful to explore the antioxidant effects of *K. coccinea* food and provide evidence that *K. coccinea* is a good source of bioactive substances, as well as screened the better varieties in terms of antioxidant activity. Subsequently, the specific composition changes at different stages can be further studied by HPLC- MS/MS, or the effects of *K. coccinea* fruits on endogenous antioxidant enzymes can be explored by animal models to further study the activity of *K. coccinea* fruits.

## Data availability statement

The original contributions presented in this study are included in the article/Supplementary material, further inquiries can be directed to the corresponding authors.

## Author contributions

XL: writing – original draft and writing – review and editing. MT: data curation and writing – original draft. YC and CJ: methodology. SH: writing – review and editing. HL: writing – review. JL: conceptualization and supervision. JR: validation and resources. All authors have read and agreed to the published version of the manuscript.
