# Supplementary figures and images for: Effects of simulated in vitro gastrointestinal digestion on antioxidant activities and potential bioaccessibility of phenolic compounds from K. coccinea fruits
Source: Front Nutr. 2022 Dec 15;9:1024651. doi: 10.3389/fnut.2022.1024651 (PMC9798096; doi:10.3389/fnut.2022.1024651)

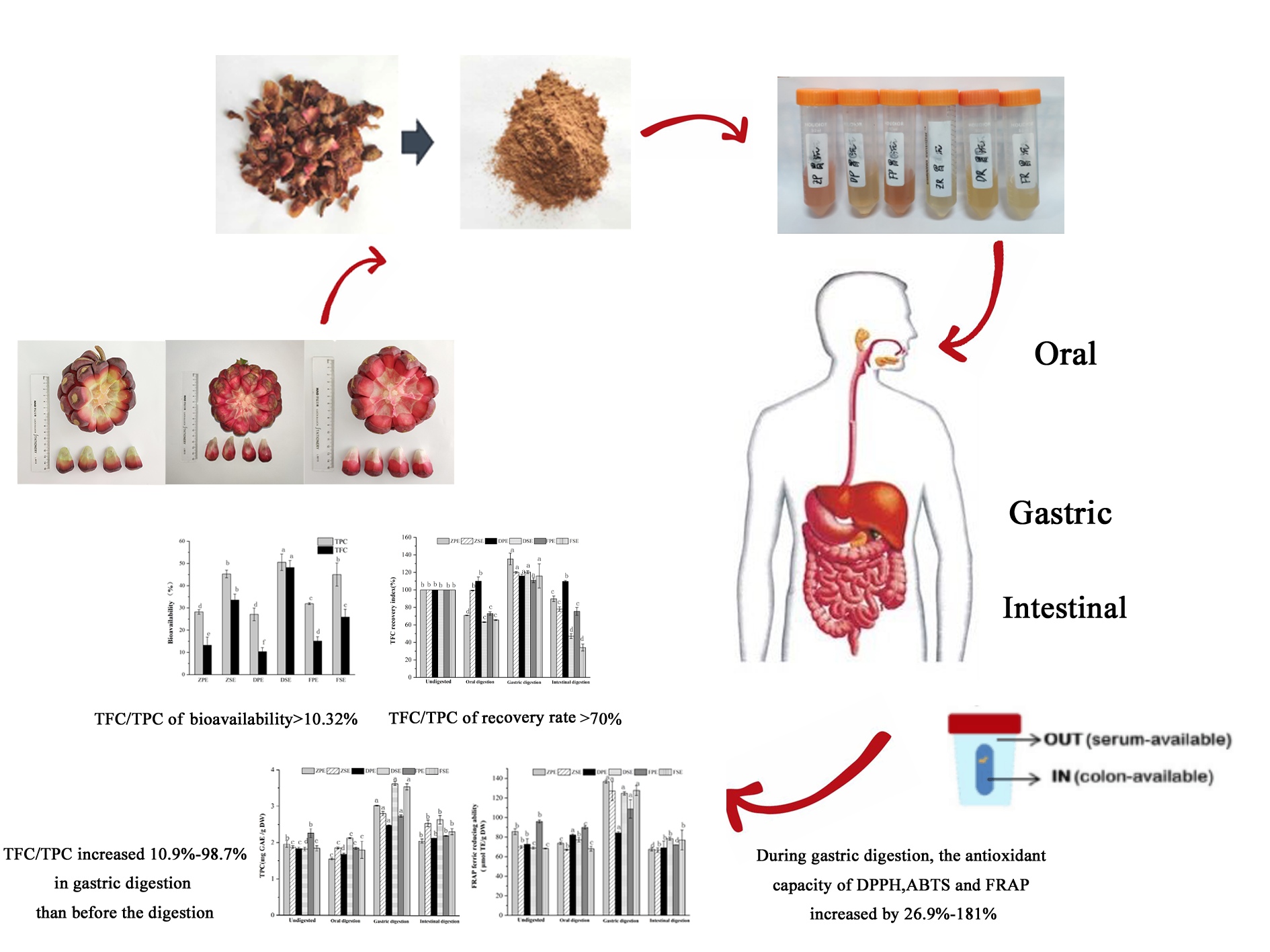

Supplement: Supplementary file 1 [file Image_1.png]
